# Laboratory data on dynamics of microplastic particles sinking in seawater with dispersed xanthan gum and kappa-carrageenan and rheological properties of these dispersions

**DOI:** 10.1016/j.dib.2024.110101

**Published:** 2024-02-02

**Authors:** Magdalena M. Mrokowska, Anna Krztoń-Maziopa

**Affiliations:** a Institute of Geophysics, Polish Academy of Sciences, Ks. Janusza 64, 01-452 Warsaw, Poland; b Warsaw University of Technology, Faculty of Chemistry, Noakowskiego St. 3, 00-664, Warsaw, Poland

**Keywords:** Rheology, Particle settling, Polysaccharide, Sedimentation, Non-Newtonian fluid, Exopolymer

## Abstract

This paper presents a dataset comprising measurements of the sinking dynamics of microplastics settling in artificial seawater (AS) and in dispersions of polymers in seawater: xanthan gum, kappa-carrageenan, and their mixtures in two concentrations: 0.5 g/L and 1 g/L. Plastic particles are classified into fifteen groups representing various shapes: disks, rods, blades, spheres, and materials: PS, POM, PET, PA6. The sinking of individual particles in a settling tank was visualized using the shadowgraph method and images were recorded using a camera with macro lenses. Next, Particle Tracking Velocimetry was applied to retrieve the time-resolved position of MPs and their orientation and to calculate instantaneous sinking velocity. Non-Newtonian properties of solutions were measured using a rheometer. Shear-dependent viscosity, shear stress amplitude sweeps, the first normal stress difference, and gelling time were assessed. Datasets may find application in a range of scientific and engineering areas including fluid mechanics, chemical engineering, food engineering, petroleum industry, wastewater treatment, rheology, and environmental hydrodynamics, e.g. in research on particle dynamics in complex fluids, modeling of microplastics fate in aqueous systems, and to develop numerical models on the hydrodynamics of solid particles in complex liquids.

Specifications Table

**Table:** 

Subject	Chemistry
Specific subject area	physical chemistry, the chemistry of colloids, rheology, two-phase flows with elements of oceanography, environmental chemistry, and pollution studies
Data format	Raw, Filtered
Type of data	.csv files (datasets with numbers and text headings) .txt files (description of dataset content)
Data collection	Particle settling experiments were performed in a tank (0.5 m high, 0.11×0.11m inner dimension) with transparent walls and backlight. Basler acA2500-60um camera with Schneider-Kreuznach macro lenses Componon 2.8/28–001 with aperture 3.5 F mounted perpendicular to the front wall of the tank was used to capture images of particles settling in test solutions. Data were recorded by digital video camera recording software StreamPix®. ImageJ and Matlab® Image Processing Toolbox® enabled data pre-processing and processing, and next the assessment of time-resolved microplastic centroid position and orientation. Samples of test solutions (about 10 ml) were taken to measure rheological properties using Physica MCR 301 rheometer with a coaxial cylinder measuring geometry and a built-in Peltier device for temperature control.
Data source location	Institute of Geophysics, Polish Academy of Sciences, Ks. Janusza 64, Warsaw, Poland Warsaw University of Technology, Noakowskiego St. 3, 00-664, Warsaw, Poland
Data accessibility	Repository name: Mendeley Data Data identification number: 10.17632/pb7fjnwcw4.2 Direct URL to data [1]: https://data.mendeley.com/datasets/pb7fjnwcw4/2 Instructions for accessing these data: open access
Related research article	[2] M.M. Mrokowska, A. Krztoń-Maziopa, Settling of microplastics in mucus-rich water column: The role of biologically modified rheology of seawater, Science of The Total Environment, 912 (2024) 168767, doi: 10.1016/j.scitotenv.2023.168767

## 1 Value of the Data

• Dataset is useful in understanding the impact of polysaccharides on the rheological properties of seawater and the effects of polymer mixtures on the rheology of electrolyte solutions.
• Dataset provides new observations on the non-Newtonian effects in settling dynamics of solid particles of various shapes.
• Data may be useful for researchers investigating physio-chemical properties of non-Newtonian fluids and settling of solid particles in these systems representing the fields of chemical engineering, rheology, food technology, petroleum engineering, pharmaceutical industry, wastewater treatment, environmental sciences, marine sciences, and environmental pollution.
• This dataset may be used to develop numerical models on sinking dynamics of regular and irregular solid particles in non-Newtonian fluids.
• The dataset may be useful from the viewpoint of marine chemistry, oceanology, and marine sciences since it presents unique measurements of seawater properties modified by natural polymers.
• Data may be used to model the fate of microplastics and other solid particles in marine waters with excessive exopolymers.

## 2 Background

This dataset was collected within laboratory research on the effects of excessive amounts of exopolymers (EPSs) on the rheology of seawater and implications for settling dynamics of microplastics (MPs) in marine environments [2]. Field studies have already demonstrated that EPSs secreted in excess form a gel-like network during algal blooms [3] that transform seawater into rheologically complex liquid [4], [5], [6]. However, the rheological effects of such modified marine systems on physical, biological, and chemical processes are not well understood [7,8] and the dataset represents significant input into this field of research. The laboratory procedure and data will help other research groups study the rheology of biologically modified seawater and MPs transport.

Moreover, the data can be used in a wide range of research and engineering problems involving complex fluids and settling dynamics of solid particles including wastewater treatment technology, food processing, petroleum industry [9]. Widely used commercial polysaccharides - xanthan gum and κ-carrageenan, have been selected in this study to demonstrate their impact on the rheological characteristics of electrolyte solutions, which is a problem of practical importance.

## 3 Data Description

The data present measurements from seven experiments on MPs settling in artificial seawater and seawater with the addition of polysaccharides: xanthan gum, κ-carrageenan, and their mixtures. In settling experiments, data on the time-resolved position of particle centroid and orientation was collected. Moreover, each test solution was measured for its rheological properties.

### 3.1 Dimensions of Particles

The dataset collected in the **Dimensions_of_particles** folder comprises dimensions of test MPs measured for samples containing a few to over one hundred particles. Thus, the folder contains 14 .csv files ***MPType_MPSub-Type*.csv**, where *MPType* indicates (S – sphere, D – disk, R – rod, B – blade, I – irregular), *MPSub-Type* is a sub-type of particle (a, b, c) if more than one kind of MP is considered in terms of dimensions for a given shape. These data were next used to calculate the average dimensions of MPs collected in Table 1. Dimensions were not measured for one particle type, S_PS, since they were provided by the manufacturer.

**Table 1:** Physical properties of test microplastic particles: length, breadth, and thickness (provided as mean values).

particle type	density (g/cm^3^)	L, length (mm)	I, breadth (mm)	S, thickness (mm)
S_PS	1.05	1.042	1.042	1.042
S_POM_a	1.37	1.51	1.50	1.50
S_POM_b	1.37	2.90	2.89	2.89
D_PET_a	1.34	1.96	1.96	0.20
D_PET_b	1.34	3.00	3.00	0.20
D_PET_c	1.34	3.03	3.03	0.30
B_PET_a	1.34	4.19	1.3	0.20
B_PET_b	1.34	3.84	2.08	0.20
R_PET_a	1.36	2.39	0.32	0.32
R_PET_b	1.36	4.05	0.3	0.30
R_PA_a	1.13	2.39	0.47	0.47
R_PA_b	1.13	3.93	0.48	0.48
I_POM	1.41	3.55	3.09	2.87
I_PA	1.13	3.35	2.89	2.48
I_PS	1.05	4.16	3.69	3.07

### 3.2 Settling Experiments

**Fig. 1:**
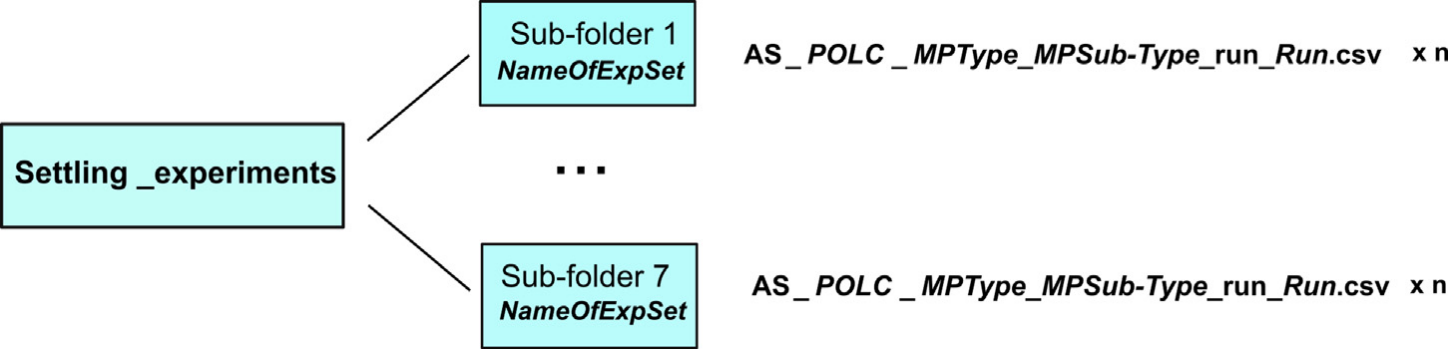
Schematic structure of data produced in settling experiments, *n* is the number of runs in an experimental set.

Dataset from settling experiments collected in the **Settling_experiments** folder comprises time-resolved horizontal, and vertical coordinates of MP centroid and time-resolved orientation of microplastic particles. Fig. 1 presents the scheme of data organization.

Each of the seven sub-folders ***NameOfExpSet*** comprises data for a separate experimental set dedicated to a test solution. Folders are named after solution type referred to as *AS_POLC* with *AS* indicating artificial seawater; *POL* indicating polymer type: *XG* – xanthan gum, *CR* – κ-carrageenan, and *XGCR* – a mixture of xanthan gum and κ-carrageenan; *C* stands for the concentration of polymer which is 0.5 or 1 g/L.

Each sub-folder contains *n* .csv files, where *n* is the number of runs in the experiment, **AS_*POLC*_*MPType_MPSub-Type*_run_*Run*.csv**, where *MPType* indicates (S – sphere, D – disk, R – rod, B – blade, I – irregular), *MPSub-Type* is a sub-type of particle (a, b, c) if more than one kind of MP is considered in terms of dimensions for a given shape (see Table 1 for definitions). R*un* denotes the number of runs for a particular *MPType* and *MPSub-Type* in the experimental set. In files, data are organized in four columns: *x [pix], z [pix], t[s]*, and *angle[degrees]* where *x and z* are horizontal and vertical coordinates of MP centroid, *t* is a time instant, and *angle* indicates an angle between the x-axis and the major axis of the ellipse that has the same second-moments as the particle projection assessed using Matlab Image Processing Toolbox®.

### 3.3 Rheology

**Fig. 2:**
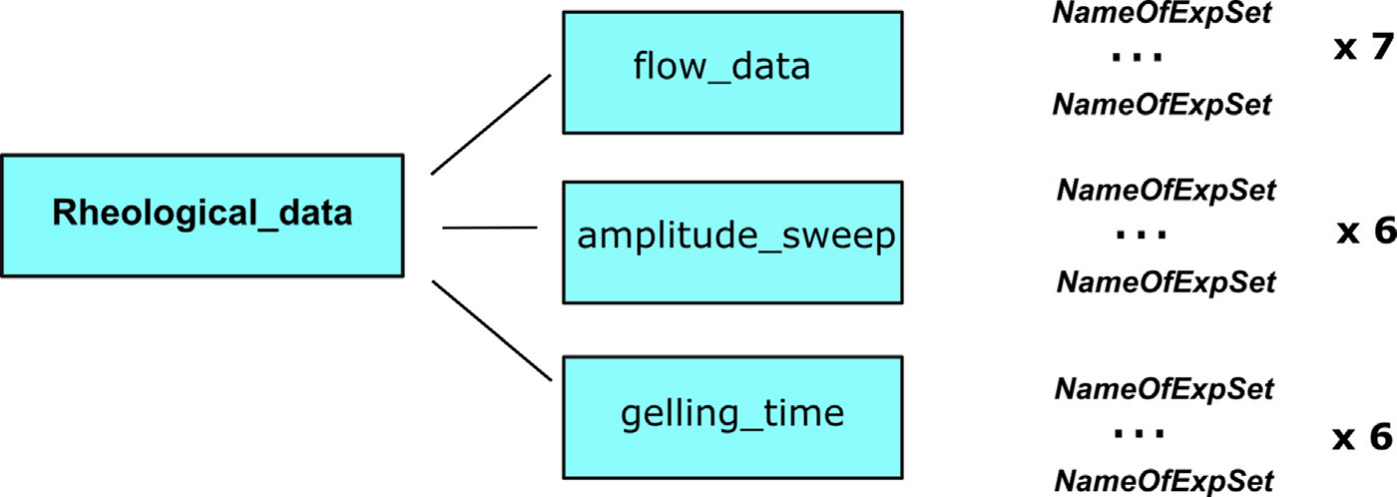
Schematic structure of rheological data.

Rheological measurements collected in **Rheological_data** comprise flow properties, viscoelasticity, and determination of gelling time. Fig. 2 presents a scheme for the organization of these data. Flow properties data are stored in the ***flow_data*** folder, viscoelasticity data in the ***amplitude_sweep*** folder, and data on gelling in the ***gelling_time*** folder. Each of these three folders contains .csv files named using the *AS_POLC* convention explained in section 1.1 with respective data for each test solution. ***Flow_data*** contains seven files with columns: *ShearRate [1/s], ShearStress [Pa], Viscosity [Pa s], Speed [1/min], Time [s], Torque [µN m],1st Norm_stress_diff [Pa]*, where Viscosity indicates dynamic viscosity, Time – time instant, *1st Norm_stress_diff* – the first normal stress difference*.* ***Amplitude_sweep*** folder contains six files (no measurements for AS solution) containing four columns: *gamma [%], tau [Pa], StorageMod [Pa], LossMod [Pa], ComplVisc [Pa s]* where *gamma* indicates strain, *tau* – shear stress, *StorageMod* – storage modulus, *LossMod* – loss modulus, *ComplVisc* – complex viscosity.

## 4 Experimental Design, Materials and Methods

Settling dynamics of fifteen groups of microplastic particles settling in artificial seawater (AS) and in dispersions of polymers in seawater: xanthan gum (XG), κ-carrageenan (CR), and their mixtures (XG+CR), were carried out and accompanied with rheological measurements of test solutions.

### 4.1 Materials

**Fig. 3:**
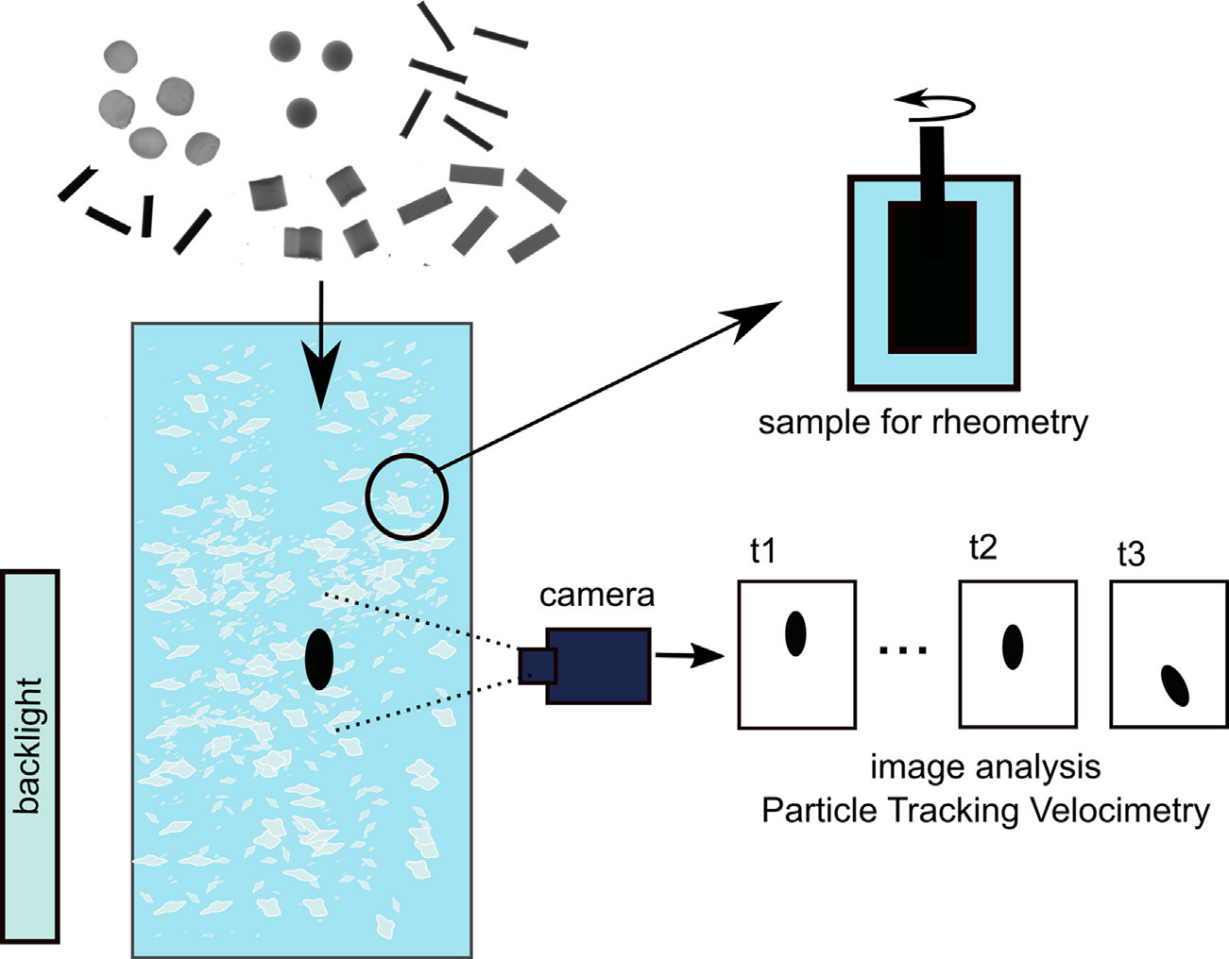
Scheme of the experimental set-up.

Seven types of solutions were prepared based on artificial seawater comprising NaCl, MgCl2, Na2SO4, CaCl2, KCl, NaHCO3, KBr, H3BO3, SrCl2, NaF. Artificial seawater was prepared according to ASTM standards [10] that specify types and amounts of salts comprising standard seawater of salinity 3.5%. Analytical-grade polysaccharides: xanthan gum (XG, Sigma Aldrich CAS: 11138-66-2) and κ-carrageenan (CR, Sigma Aldrich CAS: 11114-20-8), were used to get a solution of 0.5 g/L and 1 g/L of each polysaccharide and a mixture of the two polysaccharides in 4:1 proportion. First, polysaccharides were added to 1L of distilled water to get a well-hydrated and dissolved solution. Next, each of them was mixed with a 1L solution of salts comprising AS, and distilled water was added to obtain a designated volume of solution (Fig. 3).

Fifteen groups of particles varying in shape and material were studied in the experiments: three sets of disks, four sets of rods, two sets of blades, three sets of spheres, and three sets of irregular particles. Particles were composed of plastic materials: PA – polyamide 6, POM – polyoxymethylene, PS – polystyrene or polystyrene divinyl benzene, and PET – polyethylene terephthalate with densities and dimensions specified in Table 1. Irregular particles and spheres were industrial beads, while other shapes were manufactured in the laboratory from foils (disks and blades) and fibers (rods). In each settling experiment, a few replicate particles from the same group were tested.

### 4.2 Settling Experiments

Settling experiments were performed in the Laboratory of Hydrodynamic Micromodels, Institute of Geophysics, Polish Academy of Sciences. Experiments were performed in a transparent tank with an inner cross-section of 0.11 m x 0.11 m and a height of 0.5 m. After filling the tank with a test solution, a temperature was controlled using a liquid thermometer with an accuracy of up to 0.1 ℃ to exclude convective motions in the liquid. A series of a few replicate particles from each group were released to the tank using a tweezer. Particles were introduced beneath the surface of the test liquid one by one with at least a 3-minute interval between tests to let the solution relax.

The shadowgraph method was used to visualize sinking particles by illuminating the back of the tank with an LED panel. Images of sinking particles were acquired using a monochromatic Basler camera acA2500-60um with Schneider-Kreuznach macro lenses Componon 2.8/28–001 with aperture 3.5 F and 6 mm extension tube operating at 2590 × 2048 resolution and one pixel corresponding to 31 µm. Images were captured with frequency varying between 20 and 90 fps depending on the rate of sinking process for each particle.

Acquisition parameters were controlled using the digital video camera recording software StreamPix®. Images recorded directly on the disk of a computer were manually selected to remove any low-quality or incomplete datasets. Retained images were cropped to decrease the volume of images. Next, they were converted into a binary mode using thresholding available in Matlab®, and particles were identified in the images. In the next step, the centroid and angle (angle between the x-axis and the major axis of the ellipse that has the same second-moments as the particle projection) using Image Processing Toolbox® in Matlab® were identified.

### 4.3 Rheological Tests

Rotational rheometer Physica MCR301 was applied to measure the rheological properties of test solutions at the Department of Inorganic Chemistry of Warsaw University of Technology. Measurements were performed in a coaxial cylinder measuring geometry (CC17-SN12952, 0.714 mm gap), at a temperature of 21°C. Flow properties were captured in a controlled shear rate mode in the shear rate ranging from 0.1 to 100 s^−1^. The first normal stress difference was calculated using a modified version of the Cross equation. Amplitude sweeps were recorded in small strain oscillatory tests at a constant frequency of 1Hz with shear stress amplitude ranging from 0.00001 Pa to 20 Pa. In the gelling test, an initial shear rate of 100 s^−1^ was applied for 60 s and then the sample was relaxed for 120 s. A constant shear stress of 0.001 Pa was abruptly applied to record the resultant shear rate.

## Limitations

Not applicable.

## Ethics Statement

The authors have read and followed the ethical requirements for publication in Data in Brief and confirm that the current work does not involve human subjects, animal experiments, or any data collected from social media platforms.

## CRediT Author Statement

**Magdalena Mrokowska:** Conceptualization, Methodology of settling experiments, Execution of settling experiments, Curation of data from settling experiments and rheology measurements, Design of database, Writing - original draft preparation, Editing, Visualization, Funding acquisition, Project administration. **Anna Krztoń-Maziopa:** Methodology of rheological measurements, Curation of rheological data, Writing - description of rheological measurements, Reviewing.

## Data Availability

Dataset on rheological measurements of xanthan gum and carrageenan dispersed in seawater and settling dynamics of spherical and non-spherical microplastics in these dispersions (Original data) (Mendeley Data).
